# Dentition in the Horse and Other Animals*Read before the Annual Meeting of the Odonto-Chirurgical Society of Edinburgh.—*British Journal of Dental Science*.

**Published:** 1882-07

**Authors:** 

**Affiliations:** Edinburgh


### POPULAR SCIENCE DEPARTMENT.

### DENTITION IN THE HORSE AND OTHER ANIMALS.*

BY PRINCIPAL WILLIAMS, EDINBURGH.

After some preliminary remarks, Principal Williams said he might state, in the first place, that a horse had forty teeth—twelve incisors, four canines, and twenty-four molars. In a specimen which he held in his hand the number of teeth was found to be forty-four (the typical number), and, remarkable to relate, they were the teeth of a mare, in which, generally, the canines were wanting, or very rudimentary. When they were there, they were in the lower jaw, and, as a rule, they were not to be found in the upper jaw. But, in this case, there were two well-developed teeth above—a most remarkable circumstance, the like of which he had never seen before. One of the molars was diseased and had to be removed. The mare lived for some time after that, and he watched the case very carefully.

A foal was born with two central incisors in each jaw, and a calf had eight incisors at birth, or a few days afterward. A pig had four incisors at birth ,or a few days after. He might state here a remarkable fact. In the horse the incisors were the central teeth ; in the calf the central ones were cut first and the side ones (corner) afterward. But in the pig the corner incisors were cut first. That was a very kind arrangement of Providence, for when a pig was sucking its mother they knew how it tugged and tore at her, and if the incisors were in the centre of the mouth they would injure the lacteal parts. That was, however, prevented by there being only one incisor at each corner.

When nine weeks old the horse had four incisors, and when nine months there was the appearance presented by the very nice specimen which he now showed to them. There were in it four incisors cut and two cutting. At twelve-months old the infundibulum, or " mark " of the central teeth, was commencing to be obliterated by the wearing down of the teeth. There were no changes in the incisors of the horse till he was two and a half years old, when the central ones were shed ; at three and a half years the lateral ones ; and at four and a half years

---

* Read before the Annual Meeting of the Odonto-Chirurgical Society of Edinburgh.—*British Journal of Dental Science.*

the corner ones. At five years of age the horse was said to have a full mouth. He might state that no amount of care in the breeding of the horse, or in its feeding, made any great difference in the process of dentition. A Highland pony would have as full a mouth as a highly pampered horse. Perhaps, in some cases, where animals were very highly fed, the corner incisors might be cut a month or two sooner than if the horse was otherwise treated. But if they turned to the cow they found a remarkable thing. He had said that the calf had eight incisors. At the age of one year and nine months the central temporary incisors were shed, and the permanent ones cut. If we took an animal of the Highland, or any of the unimproved breeds, although we found that at two and a half years of age the central permanent incisors were cut, still such an animal did not have a full mouth of permanent teeth till four and a half or five years of age. On the other hand, they found that improved breeds had at two years of age four permanent incisors. At two and a half they would have six, and at two years and nine months they would sometimes have a full mouth. They were as well furnished with teeth in every possible way at the age of two years and nine months as the unimproved animals were at five years of age. The science of agriculture, while it enabled the farmers to grow two blades of grass where only one grew before, almost enabled them to raise two animals instead of one. In the same time that one unimproved animal took to arrive at maturity they would have two improved ones in the same condition.

As regarded the molars of the horse, there were at birth, or shortly afterward, three temporary molars in each jaw. At the age of one year the first permanent molar was cut, and it stood fourth in the row. When the horse was two years old the second permanent molar was erupted, and it stood fifth in the row. At three years the two anterior temporary molars were replaced by permanent ones. The sixth molar was cut, and at the same time the remaining temporary molar, which stood third in the row, was replaced by a permanent one. He had an explanation to make with reference to the fourth molar. It was the shortest molar in the mouth, and it was the tooth that was most frequently diseased. His experience was, that among the herbivora—and he might include the pig—caries of the teeth did not exist; that is, the kind that affected the human teeth. He thought that in these animals there was no caries but necrosis. This molar was a short tooth; but continued to grow almost as long as the animal lived.

he then exhibited a specimen in which the obliteration of the nutrient foramen was nearly completed ; the circulation thus cut off necrosis of the dentine resulted, and he believed that was the only actual disease there was in the herbivora that we could compare with caries. He exhibited a specimen which had become soft, and, as a result, splits had taken place in the tooth. In consequence of that the food had worked up into the cavities of the face, and, as a result, there were great dischares from the nostrils. One of the symptoms of a diseased tooth was a fetid discharge from the nose, continuing often for a long period. If, by examination, portions of food could be found in the discharge from the nose, this would point conclusively to the fact that a cavity had been formed in the tooth which admitted of the entrance of the food into the facial sinuses.

The dog had forty-two teeth, and the arrangement was not quite so symmetrical. In the dog caries was found as true as it was found in the human being.

Then, as to camel's teeth, he had several specimens which he had received from India ; but he had little to say with regard to them. It would be observed that in the upper jaw there were only two incisors, as they were termed by some writers on anatomy ; but they appeared to him to be more like canines. There were only two canines and two incisors. It was a remarkable arrangement that in the ruminating animals the incisors were in the lower jaw only.

A beautiful arrangement existed with reference to the ox's teeth. They were fitted in quite an elastic way, and not fixed. When the chisel-like teeth were made use of in the act of grazing, there was an elastic cushion which prevented fracture of the teeth. He never saw a diseased tooth in the cow, and broken teeth very rarely. Notwithstanding the force with which a cow grazed in a field, and the liability of coming into contact with stones, it seldom happened that the teeth were broken, owing, no doubt, to the fact that they were on an elastic cushion. Returning to dentition, he might mention that young oxen—two or three years of age—sometimes began to lose flesh, and many of them were condemned all over the country as suffering from chronic disease, and very often destroyed. The majority of these cases were suffering from the crown of the temporary teeth remaining in the gum and preventing the animal from masticating its food. (Several specimens of these were shown.) These animals were supposed to be in a dying condition, and he had found in many cases these teeth sticking into the gums, and preventing the animal from eating. There was a man in the north of England who followed the profession of an animal dentist. He went about and examined the cattle, and gave them relief at once —by removing the portions of the teeth that were troubling them.

The Principal then exhibited a specimen showing the formation of a tumor, and several scores of teeth in a cavity. They would notice that the teeth were very imperfect. He had seen tumors of that kind with 500 imperfectly developed teeth. It would be very interesting to be able to explain how these were formed. He had seen two or three cases of the kind, and the late Professor Dick had a specimen containing an immense number of these imperfectly developed teeth. He had also seen teeth growing from the petrous portion of the temporal bone, and he had removed from one animal three of them consecutively one year after another. It was a most remarkable thing, and very difficult to account for. He had also seen teeth in testicles, and various parts of the body, enclosed in little tumors. These teeth were generally very hard. The teeth from the petrosa were excessively hard. They were most difficult to remove, requiring very great force before they could be detached from the surroundings, very often breaking, and breaking with a fracture like ivory. They seemed to be very hard dentine ; and why they should be there was beyond his comprehension. He exhibited a specimen, and pointed out that the gum came very near the crown of the teeth. With regard to the removal of the molars, it might be observed that the proportion of teeth exposed was very short ; and when they took into consideration the length of the fang, as shown in the specimen now exhibited, they would understand how extremely difficult the removal of a tooth became. He had, however, removed them without any difficulty whatever by trephining. He thought that it was by far the most humane way ; for, however strong the forceps might be, there was very great danger of fracturing the jaw, owing to the great length of the fang. It was difficult to get anything to grasp the molar. They could not fix the mouth of the horse in the same way that they could fix the mouth of a human being. Then, if they used chloroform, or any anæsthetic, there was great risk of the animal being suffocated by the hemorrhage. He had trephined the lower jaw, and he considered it was the most expeditious and best way of dealing with such cases. He then exhibited a specimen showing disease of the facial bone (osteoporosis), and where the teeth were quite healthy. It was an animal five years of age. That was not an uncommon disease, and it had been increasing of late years. He had seen more of it since foreign horses had been brought into the country. In this disease the skeleton became excessively brittle. He had a specimen showing that twenty-four ribs of a horse had been fractured, some of them in three or four places. It was remarkable that, although the bones in the face were the first to suffer, the horse might continue to have the disease, and still the teeth did not suffer. He then exhibited two cases of teeth, each of which seemed as if it contained another within it. He had only seen those two cases in all his life. In his concluding remarks, Principal Williams said that he should have been glad if he had thrown any light on the disease of the teeth. If the information he had given as to the absence of caries in the herbivora—if the information he had given, that after a long experience he had not met with what he considered pure caries in those animals, would throw some light on the question of food, or something else which might be the cause of caries in the human being—he should be highly gratified. He begged to thank the Society for the attention with which they had listened to him.

